# Asymmetry of Lacunae between Brain Hemispheres Is Associated with Atherosclerotic Occlusions of Middle Cerebral Artery

**DOI:** 10.3390/brainsci13071016

**Published:** 2023-06-30

**Authors:** Lingshan Wu, Hao Huang, Zhiyuan Yu, Xiang Luo, Shabei Xu

**Affiliations:** Department of Neurology, Tongji Hospital, Tongji Medical College, Huazhong University of Science and Technology, Wuhan 430030, China

**Keywords:** intracranial atherosclerotic stenosis, cerebral small vessel disease, lacunae, white matter hyperintensities

## Abstract

Cerebral small vessel disease (CSVD) commonly coexists with intracranial atherosclerotic stenosis (ICAS). Previous studies have tried to evaluate the relationship between ICAS and CSVD; however, they have yielded varied conclusions. Furthermore, the methodology of these studies is not very rigorous, as they have evaluated the association between ICAS and CSVD of bilateral hemispheres rather than the affected hemisphere. Unilateral middle cerebral artery atherosclerotic occlusion (uni-MCAO) is a favorable model to solve this problem. Material and methods: Patients with uni-MCAO were retrospectively observed. Imaging characteristics, including lacunae, white matter hyperintensities (WMH), enlarged perivascular spaces (EPVS), and cerebral microbleeds (CMBs), were compared between the hemisphere ipsilateral to the MCAO and the contralateral hemisphere. Results: A total of 219 patients (median age 57 years; 156 males) were enrolled. Compared with the contralateral side, increased quality of lacunae (median, IQR, 0, 2 vs. 0, 1; *p* < 0.001) and elevated CSVD score (median, IQR, 0, 1 vs. 0, 1; *p* = 0.004) were found in the occluded hemisphere. No significant differences were shown for WMH, EPVS, and CMBs. Conclusions: Uni-MCAO has a higher prevalence of lacunae in the ipsilateral hemisphere. However, no interhemispheric differences in WMH, EPVS, or CMBs were found.

## 1. Introduction

Intracranial atherosclerotic stenosis (ICAS) is one of the most common causes of stroke worldwide [1], which may lead to hypoperfusion and damage to the downstream small vessel bed [2]. Diseases affecting small vessels in the brain are implicated in the development of chronic cerebral small vessel disease (CSVD) [3]. Thus, they may share common imaging characteristics, such as recent small subcortical infarcts, lacunae, white matter hyperintensities (WMH), and even enlarged perivascular spaces (EPVS), cerebral microbleeds (CMBs), and brain atrophy [4], all of which were traditionally considered typical imaging markers for CSVD. Previous studies have indicated that ICAS and CSVD frequently coexist in different neurological diseases, such as cerebrovascular ischemic events, dizziness, and so on [1,5,6]. However, the underlying pathological mechanism association between ICAS and CSVD is not fully understood. Thus, studying the correlation between ICAS and CSVD may provide a theoretical basis for a better understanding of the mechanisms of CSVD.

Several studies have evaluated the relationship between ICAS and CSVD. In one conclusion, Du et al. found a biological link between a severe steno-occlusive middle cerebral artery (MCA) and increased basal ganglia EPVS [7]; Park et al. found that ICAS is independently associated with progressively greater WMH burden [8]; Zhai et al. found that ICAS seems strongly associated with lacunae and WMH, but not CMBs or EPVS [9]. Conflicting results and variable conclusions were addressed. Furthermore, the trial design was not very rigorous, because most of these studies analyzed the association between ICAS and CSVD in bilateral hemispheres, which may cause errors as the contralateral hemisphere is not affected by ICAS. Thus, more rigorous research should be conducted to analyze the relationship between ICAS and CSVD.

Unilateral intracranial atherosclerotic lesions can avoid this problem. Therefore, we included patients with unilateral MCA atherosclerotic occlusion (uni-MCAO) in the present study, recorded their imaging characteristics, and history information, and then, compared the imaging features of CSVD between the ipsilateral and contralateral sides. This should be an ideal model for comparing the imaging heterogeneity between ICAS and CSVD.

## 2. Materials and Methods

### 2.1. Subjects

We retrospectively reviewed the demographic characteristics, laboratory results, and imaging of patients with uni-MCAO in Tongji Hospital from January 2016 to July 2021. Inclusion criteria: (1) age >18 years; (2) with uni-MCAO in the M1 segment (the MCA stem and initial short segments of the MCA divisions), which was confirmed by computed tomographic angiography or digital subtraction angiography; (3) <50% stenosis in the internal carotid artery.

**Figure 1:**
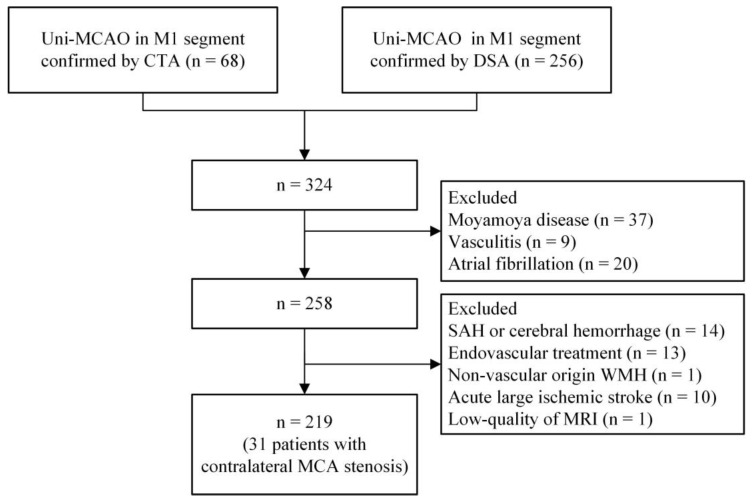
Flowchart of patient selection. Abbreviations: uni-MCAO, unilateral middle cerebral artery atherosclerotic occlusion; CTA, computed tomographic angiography; DSA, digital subtraction angiography; CSVD, cerebral small vessel disease; SAH, subarachnoid hemorrhage; WMH, white matter hyperintensities; MRI, magnetic resonance imaging.

The exclusion criteria were as follows: (1) MCA occlusion associated with Moyamoya disease, vasculitis, atrial fibrillation, or etiologies other than atherosclerosis; (2) patients had undergone endovascular treatment; (3) MCA occlusion combined with acute subarachnoid hemorrhage or cerebral hemorrhage; (4) WMH caused by non-vascular diseases, e.g., multiple sclerosis; (5) patients with acute large ischemic stroke (with a diameter above 20 mm) that could have affected the assessment of CSVD imaging markers on magnetic resonance imaging (MRI); (6) low-quality of MRI. The flowchart of patient selection is shown in Figure 1.

This study was approved by the ethics board of Tongji Hospital (No. TJ-IRB20210107), and the requirement of informed consent was waived owing to the retrospective nature of the study.

### 2.2. Clinical Data

Clinical information, including age, gender, symptomatic status (transient ischemic attack or acute infarction), hypertension, diabetes, hyperlipidemia, prior stroke, and history coronary heart disease, was collected. Smoking and alcohol consumption were also recorded. The following laboratory results at admission were collected: levels of triglycerides, high-density lipoprotein, low-density lipoprotein, total cholesterol, urea, creatinine, and cystatin C; and estimated glomerular filtration rate (eGFR). eGFR was calculated using the Chronic Kidney Disease Epidemiology Collaboration equation [10].

### 2.3. CSVD Assessment

MRI markers of CSVD, including EPVS, WMH, CMBs, brain atrophy, and lacunae, were assessed by two experienced observers blinded to clinical information. Disagreements were resolved through discussion, and when necessary, a third reader with expertise in the field was consulted.

According to the Standards for Reporting Vascular Changes on Neuroimaging criteria [4], a lacuna was defined as a rounded or oval lesion, >3 mm and <15 mm in diameter, in the region of the basal ganglia or centrum semiovale, with a signal similar to cerebrospinal fluid. We accessed the number of lacunae in the basal ganglia and centrum semiovale on the ipsilateral and contralateral sides. 

WMH was defined as hyperintense in T2-weighted and fluid-attenuated inversion recovery images and isointense or hypointense in T1-weighted images. To assess the severity of WMH, we applied the age-related white matter changes scale (ARWMC scale, which rated the frontal area, parieto-occipital area, temporal area, infratentorial area, and basal ganglia WMH in the right and left hemispheres separately; grades 0–30) [11] and van Swieten scale (VSS, which quantified anterior and posterior periventricular WMH of the whole brain; grades 0–4) [12]. For patients with acute diffusion-weighted imaging lesion(s), the evaluation was conducted more carefully to avoid considering infarct lesions in the corresponding fluid-attenuated inversion recovery image as WMH.

EPVS were defined as fluid-filled spaces that follow the typical course of a vessel as it traverses grey or white matter. EPVS was assessed in the region of basal ganglia based on the following visual rating scale: grade 0, no EPVS; grade 1, 1–10 EPVS; grade 2, 11–20 EPVS; grade 3, 21–40 EPVS; and grade 4, >40 EPVS [13]. 

CMBs were defined as rounded homogeneous lesions (2–10 mm in diameter) with low signal intensity on susceptibility-weighted imaging sequences [4]. We accessed the number of CMBs on the ipsilateral and contralateral sides.

We defined brain atrophy as cortical and deep; the severity of cortical or deep atrophy was rated as none, mild–moderate, and severe against a reference brain template, yielding a 5-point ordinal scale (0–4, where 0 means no atrophy, and 4 means most severe atrophy) [14].

To assess the CSVD scores, we applied a scale that combines individual MRI features (WMH, lacunae, and brain atrophy) [14]. CSVD scores ranged from 0 to 3 (0, no disease; 3, most severe disease), representing several different CSVD types. The presence of each of the markers was awarded 1 point for the following: (1) severe white matter changes (VSS score ≥3); (2) number of lacunae ≥2; and (3) severe brain atrophy (atrophy score ≥3). 

### 2.4. Collateral Grading

Collateral vessel status was assessed by two experienced observers who were blinded to clinical information and MRI markers of CSVD, using the ASITN/SIR scale [15] for digital subtraction angiography or the Tan scale [16] for computed tomographic angiography. 

The ASITN/SIR scale was graded as 0 or 1, 2, 3 or 4; good collaterals were defined as ASITN/SIR scores of 3–4, moderate collaterals were defined as an ASITN/SIR score of 2, and poor collaterals were defined as ASITN/SIR scores 0–1 [15]. Collateral vessel scores were categorized into Tan grades 0 or 1, 2, or 3, where poor collaterals were defined as Tan scores 0–1, and good collaterals were defined as Tan scores 2–3 [17].

### 2.5. Statistical Analysis

Clinical and neuroimaging characteristics are presented as mean with standard deviation and/or median with interquartile range (IQR) for continuous variables and compared using independent sample *t*-tests or Mann–Whitney U tests. Frequencies and/or proportions were used for categorical variables, and an χ^2^ test was used to determine significant differences among groups. Univariate logistic regression analysis was applied to identify possible factors correlated with the interhemispheric difference in the number of lacunae and the CSVD scores, and variables with a *p*-value of < 0.1 were entered into multivariate logistic regression analysis. All statistical analyses were performed with SPSS version 22.0 (SPSS Inc., Chicago, IL, USA), and a two-tailed *p* < 0.05 was considered statistically significant.

## 3. Results

### 3.1. Clinical Characteristics of the Patients

According to the enrollment criteria, 66 subjects were excluded due to MCA occlusion associated with etiologies other than atherosclerosis, including 37 subjects with Moyamoya disease; 9 subjects with vasculitis; and 20 patients with atrial fibrillation. Fourteen patients were excluded due to subarachnoid hemorrhage or cerebral hemorrhage. Thirteen patients were excluded due to endovascular treatment. One patient was excluded due to WMH caused by non-vascular disease. Ten patients were excluded due to acute large ischemic stroke. One patient was excluded due to the low quality of the MRI. In the end, 219 subjects were enrolled in this study, including 31 patients with contralateral MCA stenosis (>50% stenosis). Characteristics of the study population are presented in Table 1. The median age of the study population was 57 (49–63) years, 156 (71.2%) patients were male, and 162 (74%) patients were symptomatic (including 159 acute ischemic stroke and 3 transient ischemic attacks). Hypertension was present in 152 (69.4%) patients, diabetes in 59 (26.9%), hyperlipidemia in 41 (18.7%), prior stroke in 41 (18.7%), coronary heart disease in 11 (5%), current smoking in 103 (47%), and current drinking in 75 (34.2%) patients. Poor collaterals were detected in 37 (16.9%) subjects.

### 3.2. Interhemispheric Differences in Small-Vessel Neuroimaging Characteristics

**Figure 2:**
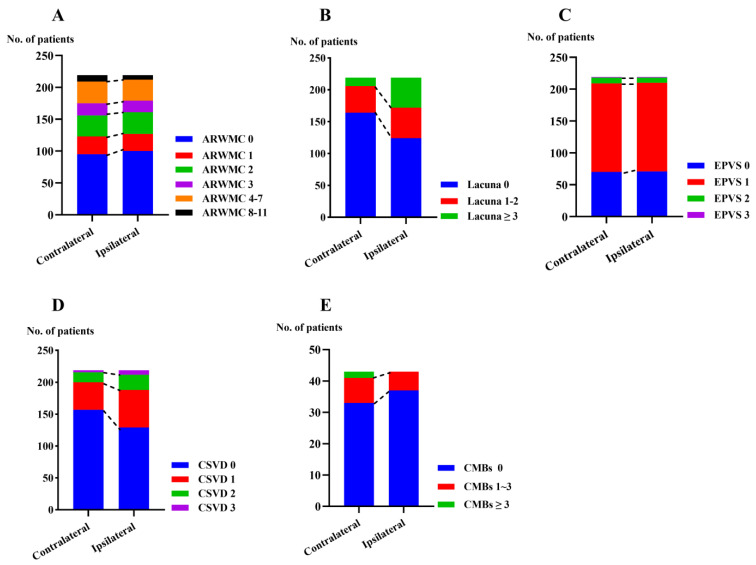
Interhemispheric differences in neuroimaging features of CSVD. Note: (**A**) Interhemispheric differences in WMH. (**B**) Interhemispheric differences in lacunae. (**C**) Interhemispheric differences in EPVS. (**D**) Interhemispheric differences in CSVD scores. (**E**) Interhemispheric differences in CMBs. Abbreviations: ARWMC, age-related white matter changes scale; EPVS, enlarged perivascular spaces; CSVD, cerebral small vessel disease; CMBs, Cerebral microbleeds.

**Figure 3:**
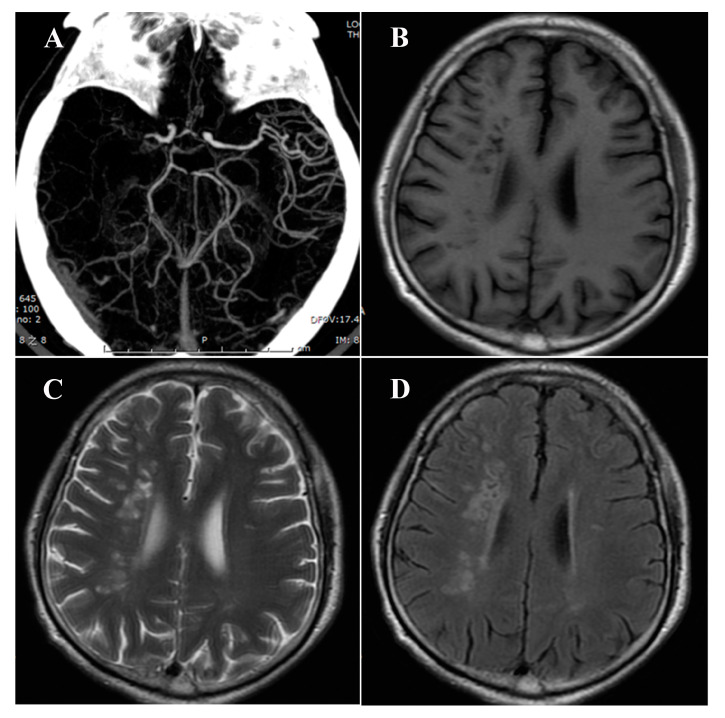
One representative case of interhemispheric differences of CSVD. Note: (**A**) Computed tomographic angiography shows occlusion of the right MCA in the M1 segment. (**B**–**D**) T1-weighted, T2-weighted, and T2-weighted fluid-attenuated inversion recovery images show increased lacuna number in the hemisphere ipsilateral to the MCAO compared with the contralateral hemisphere.

As shown in Table 2 and Figure 2, the hemisphere ipsilateral to the MCA atherosclerotic occlusion showed a significantly higher number of lacunae (43.4% vs. 25.1%, *p* < 0.001), as well as a higher ratio of patients with two or more lacunae (29.7% vs. 9.6%, *p* < 0.001), compared to the contralateral hemisphere. The numbers of lacunae (median, IQR, 0, 2 vs. 0, 1; *p* < 0.001) and CSVD score (median, IQR, 0, 1 vs. 0, 1; *p* = 0.004) in the hemisphere ipsilateral to the MCA atherosclerotic occlusion were higher than those in the contralateral hemisphere. However, no interhemispheric differences in WMH or EPVS were found. Figure 3 shows interhemispheric differences in CSVD in one representative patient.

As shown in Table 3, the interhemispheric differences in small-vessel neuroimaging characteristics were also compared in patients with contralateral MCA stenosis. However, no difference was found.

Of the 219 patients included, we analyzed the distribution of CMBs in 42 patients with susceptibility-weighted imaging sequences (Table 4). However, no interhemispheric differences in CMBs were found (median, IQR, 0, 0 vs. 0, 0; *p* = 0.246), as shown in Figure 2E.

### 3.3. Association of Demographics with the Heterogeneity of CSVD between Hemispheres

As shown in Table 5, in the multivariate analysis, diabetes and cystatin C were significantly associated with the interhemispheric difference in the number of the lacuna (OR = 2.03, *p* = 0.038; OR = 5.73, *p* = 0.024) and CSVD score (OR = 2.22, *p* = 0.033; OR = 9.67, *p* = 0.008).

## 4. Discussion

This study provides a favorable model to observe the association between ICAS and CSVD. Compared with the contralateral side, a higher prevalence of lacunae was located in the MCAO hemisphere in patients with uni-MCAO. However, in patients with contralateral MCA stenosis, no interhemispheric difference was found, which reaffirms the association between atherosclerosis and CSVD. Furthermore, we found that previous histories with diabetes and cystatin C were associated with the heterogeneity of lacunar number and CSVD scores between hemispheres.

The potential mechanism underlying the association between MCA atherosclerotic occlusion and lacunae might be artery-to-artery thromboembolic events caused by vulnerable plaques. If the atheroma in the MCA is positioned at the opening of its penetrating branches, it could lead to an acute occlusion of one or several penetrating arteries, thus causing a lacunar infarct [5]. There have been several studies showing that lacunae are sequelae of previous asymptomatic stroke events [18,19]. Carotid atherosclerosis is associated with silent lacunae, which suggests that small subcortical infarcts can occur via artery-to-artery embolism [20,21]. Over time, small subcortical infarcts can disappear and cavitate, eventually forming lacunae [22]. Similar to our study, a meta-analysis of cross-sectional studies showed that ICAS was found to be associated with silent brain infarction [23].

The association between MCA atherosclerotic occlusion and other imaging markers of CSVD was also evaluated in this study. An insufficient blood supply due to vascular pathology is thought to be the most important cause of WMH [24]. However, our findings showed that MCA atherosclerotic occlusion was associated with the increased presence and number of lacunae on the ipsilateral side, but not with WMH. A possible explanation is that patients with MCA atherosclerotic occlusion are more prone to embolism due to unstable plaques. Consistent with our opinion, Prabhakaran et al. found that a multiple-infarct pattern, attributed to artery-to-artery embolism, is associated with early recurrent infarcts in patients with ICAS [25]. While the imaging biomarker of abnormal perfusion did not increase the risk of recurrent infarcts after adjusting for hypertension, diabetes, and stenosis location [25]. Previous studies tried to evaluate the relationship between ICAS and WMH; however, they have yielded varied conclusions [23,26]. Similar to our study, Ammirati et al. found that there was no association between carotid atherosclerotic plaques and ipsilateral WMH [27]. However, the assessment of WMH was quantified through visual semiquantitative scales in this study, which may have caused certain evaluation deviations. To overcome these limitations, especially in the context of clinical studies involving a high number of subjects, automated and semiautomated methods that allow reliable and effective WMH segmentation and quantification should be used [28]. 

The absence of association between MCA atherosclerotic occlusion and the interhemispheric difference in EPVS implies a differential pathophysiological mechanism behind lacunae and EPVS. PVSs are generally considered to be fluid-filled compartments surrounding the small vessels in the brain, which act as a conduit for fluid transport, exchange between cerebrospinal fluid and interstitial fluid, and clearance of waster products by forming a network of spaces around cerebral small vessels [29]. Hence, it may lead to the accumulation of brain waste, if any function abnormal of EPVS or related clearance systems [30]. PVS are commonly microscopic, and not visible on conventional imaging, but they tend to increase in number and diameter with the process of aging [4,30]. Furthermore, recent studies reported that EPVS are associated with hypertension, other CSVD markers, vascular dementia, Alzheimer’s disease, cerebral amyloid angiopathy, and so on [29,31,32]. Studies have shown that inflammation involves the pathophysiological mechanisms underlying EPVS [29]. Pro-inflammatory markers and the aggregation of inflammatory cells in PVS lead to remodeling and alterations in fluid clearance [29]. Similar to our study, Shen et al. found that the grade of basal ganglia EPVS made no difference between the hemisphere ipsilateral to ICAS and the contralateral hemisphere [33].

CMBs are small round or ovoid hypointense foci with a blooming effect on MRI, best seen with susceptibility-weighted imaging [4,34]. They are most commonly located in the cortico-subcortical junction and deep gray or white matter in the brain (including cerebral hemispheres, brainstem, and cerebellum) [4]. Histopathological studies have demonstrated that CMBs contain hemosiderin deposits most likely resulting from the rupture of small arteries, arterioles, and/or capillaries [35,36]. The location of CMBs corresponds to two different types of pathogenesis. Deep CMBs are attributed to hypertensive vasculopathy, whereas strictly lobar CMBs appear to result from cerebral amyloid angiopathy [37]. Similar to our study, previous studies have investigated the relationship between ICAS and CMBs, suggesting the lack of a relationship between both [9,38].

In our study, we found that diabetes is associated with increased lacuna in atherosclerosis, which is consistent with previous research [39]. Blood–brain barrier dysfunction is thought to play a role in this relationship, which is associated with the leakage of plasma proteins and the initiation of inflammatory responses [40,41]. Cystatin C, a cysteine proteinase inhibitor, is expressed constitutively from all nucleated cells. It is proposed as a marker of kidney function [42]. Consistent with previous studies [43,44,45], our research found that cystatin C level is associated with CSVD score. The similarities between the glomerular and cerebral microvascular systems may explain this association [46].

For patients with MCA atherosclerotic occlusion, cerebral hypoperfusion secondary to flow-limiting stenosis is another problem that cannot be ignored. Collateral circulation refers to the pre-existing vascular channels that compensate cerebral blood flow when principal vascular failures [47]. In our study, we did not find any association between the poor collateral and the interhemispheric difference in lacunae. Consistent with our study, a previous study on stroke showed that there was no association between collateral flow state and imaging markers of CSVD in anterior-circulation acute ischemic stroke treated by mechanical thrombectomy [48]. However, the sample size in this study was small, and the collateral status was assessed by a conventional method. Further research, with a more accurate estimate of collateral status, is needed to assess the relationship between collateral circulation and CSVD.

## 5. Strengths and Limitations

The strengths of our study include the rigorous trial design. Namely, we only included patients with atherosclerotic occlusion, while occlusion due to reasons other than atherosclerosis was excluded. Additionally, we also found a significant difference in the lacunar number and CSVD score between the ipsilateral and the contralateral sides, which demonstrates a powerful advantage to the current study design. Previous studies tried to evaluate the relationship between atherosclerotic and CSVD; however, these studies ignore the heterogeneity of bilateral hemispheres. As the contralateral hemisphere is not affected by ICAS. By using this approach, the impact of atherosclerosis on lacunae is independent of demographic and vascular risk factors, which are shared by small- and large-vessel diseases. Therefore, the asymmetry of the imaging markers of CSVD is independently associated with unilateral cerebral artery atherosclerosis.

Our study had some limitations. First, the sample size in this study was small, and this study was a single-center study. Second, CMBs were assessed in only 42 patients, because most of the patients did not have susceptibility-weighted imaging sequences or gradient echo sequences. Third, both imaging markers of CSVD were accessed by traditional visual assessment, which may cause certain evaluation deviations. A prospective and longitudinal follow-up study may help to demonstrate if there is a causality. 

## 6. Conclusions

In conclusion, we found that uni-MCAO has a higher prevalence of CSVD in the ipsilateral hemisphere. Of the CSVD types, atherosclerosis was associated with lacuna but not WMH, CMBs, or EPVS. Further research is needed to elucidate the mechanism between lacuna and atherosclerosis.

## Figures and Tables

**Table 1:** Characteristics of patients with uni-MCAO.

Demographic Characteristics	Patients (*n* = 219)
Age, years	57 (49~63)
Male, n (%)	156 (71.2%)
Symptomatic, n (%)	162 (74.0%)
Vascular risk factors	
Hypertension, n (%)	152 (69.4%)
Diabetes, n (%)	59 (26.9%)
Hyperlipidemia, n (%)	41 (18.7%)
Prior stroke, n (%)	41 (18.7%)
CHD, n (%)	11 (5.0%)
Current smoker, n (%)	103 (47.0%)
Current drinker, n (%)	75 (34.2%)
Laboratory parameters	
TC, mg/dL	3.54 (2.99~4.25)
LDL, mg/dL	2.12 (1.66~2.80)
HDL, mg/dL	0.96 (0.83~1.08)
Triglycerides, mg/dL	1.22 (0.96~1.68)
Urea, mmol/L	4.8 (4~5.72)
Creatinine, µmol/L	73.83 ± 16.67
Cystatin C, mg/L	0.98 (0.88~1.14)
eGFR, mL/min/1.73 m^2^	93.88 ± 15.53
Collateral status, n (%)	
Poor collateral, n (%)	37 (16.9%)

Abbreviations: uni-MCAO, unilateral middle cerebral artery atherosclerotic occlusion; CHD, coronary heart disease. Note: Symptomatic, transient ischemic attack patients or acute ischemic stroke patients (confirmed by MRI).

**Table 2:** Comparison of the small-vessel neuroimaging characteristics between the hemisphere ipsilateral to MCA occlusion and the contralateral hemisphere in patients with uni-MCAO (n = 219).

CSVD Features	Contralateral Hemisphere	Ipsilateral Hemisphere	*p* Value
WMH			
ARWMC score, median (IQR)	1 (0~3)	1 (0~3)	0.508
Lacunae			
Presence of lacunae, n (%)	55 (25.1%)	95 (43.4%)	**<0.001**
Two or more lacunae, n (%)	21 (9.6%)	43 (29.7%)	**<0.001**
No. of lacunae, median (IQR)	0 (0~1)	0 (0~2)	**<0.001**
EPVS			
EPVS grade, median (IQR)	1 (0~1)	1 (0~1)	0.872
CSVD scores			
CSVD score, median (IQR)	0 (0~1)	0 (0~1)	**0.004**

Abbreviations: WMH, white matter hyperintensities; ARWMC, age-related white matter changes scale; EPVS, enlarged perivascular spaces; CSVD, cerebral small vessel disease; IQR, interquartile range. Note: *p*-values < 0.05 are marked in bold. The brain atrophy score assesses the atrophy whole brain and the van Swieten scale assesses WMH of the whole brain, without interhemispheric differences, so it is not shown in this table.

**Table 3:** Comparison of the small-vessel neuroimaging characteristics of the ipsilateral hemisphere between patients with contralateral MCA stenosis (n = 31).

CSVD Features	Contralateral Hemisphere	Ipsilateral Hemisphere	*p* Value
WMH			
ARWMC score, median (IQR)	0 (0~3)	1 (0~2)	0.75
Lacunae			
Prescence of lacunae, n (%)	11 (50%)	11 (50%)	1
Two or more lacunae, n (%)	4 (12.9%)	9 (29%)	0.119
No. of lacunae, median (IQR)	0 (0~1)	0 (0~2)	0.698
EPVS			
EPVS grade, median (IQR)	0 (0~0)	0 (0~0)	0.471
CSVD			
Total CSVD score, median (IQR)	0 (0~1)	0 (0~1)	0.429

Abbreviations: WMH, white matter hyperintensities; ARWMC, age-related white matter changes scale; EPVS, enlarged perivascular spaces; CSVD, cerebral small vessel disease; IQR, interquartile range. Note: The brain atrophy score assesses the atrophy whole brain and the van Swieten scale assesses WMH of the whole brain, without interhemispheric differences, so it is not shown in this table.

**Table 4:** Comparison of CMBs between the hemisphere ipsilateral to MCA occlusion and the contralateral hemisphere in patients, using susceptibility-weighted imaging sequences (n = 42).

CSVD Features	Contralateral Hemisphere	Ipsilateral Hemisphere	*p* Value
No. of CMB	0 (0~0)	0 (0~0)	0.246

Abbreviations: CMBs, cerebral microbleeds.

**Table 5:** Logistic regression analyses between possible predictors and interhemispheric difference in the neuroimaging features of CSVD.

Variables	No. of Lacunae				CSVD Score			
	Univariate Analysis		Multivariate Analysis		Univariate Analysis		Multivariate Analysis	
	OR (95%CI)	*p* Value	OR (95%CI)	*p* Value	OR (95%CI)	*p* Value	OR (95%CI)	*p* Value
Age	1.025 (0.996~1.055)	0.086			1.022 (0.989, 1.056)	0.188		
Male sex	0.658 (0.348~1.241)	0.196			0.613 (0.292, 1.29)	0.197		
Symptomatic	0.906 (0.483~1.697)	0.757			0.909 (0.449, 1.842)	0.791		
Poor collateral	1.15 (0.553~2.389)	0.708			0.892 (0.38, 2.095)	0.793		
Hypertension	1.16 (0.632~2.13)	0.633			1.218 (0.607, 2.442)	0.579		
Diabetes	2.046 (1.11~3.772)	**0.022**	2.029 (1.039~3.961)	**0.038**	1.9 (0.973,3.711)	0.060	2.219 (1.067, 4.615)	**0.033**
Hyperlipidemia	0.719 (0.344~1.506)	0.382			0.627 (0.26, 1.515)	0.300		
Prior stroke	1.393 (0.695~2.79)	0.350			1.475 (0.689, 3.156)	0.317		
CHD	0.174 (0.022~1.383)	0.198			0.316 (0.039, 2.53)	0.278		
Current smoker	1.876 (1.07~3.288)	**0.028**			2.301 (1.207, 4.385)	**0.011**		
Current drinker	1.786 (1.002~3.182)	**0.049**			2.269 (1.195, 4.308)	**0.012**		
TC	0.945 (0.712~1.255)	0.695			0.885 (0.638, 1.226)	0.461		
LDL	0.937 (0.676~1.299)	0.696			0.878 (0.604, 1.277)	0.496		
HDL	1.101 (0.302~4.009)	0.884			1.209 (0.284, 5.149)	0.798		
Triglycerides	0.848 (0.59~1.221)	0.376			0.642 (0.374, 1.103)	0.109		
Urea	0.99 (0.95~1.032)	0.627			0.993 (0.957, 1.03)	0.692		
Creatinine	1.012 (0.995~1.029)	0.176			1.017 (0.998, 1.036)	0.079		
Cystatin C	4.94 (1.109~21.996)	**0.036**	5.725 (1.252~26.173)	**0.024**	8.042 (1.554, 41.613)	**0.013**	9.674 (1.801, 51.969)	**0.008**
eGFR	0.985 (0.967~1.003)	0.107			0.982 (0.962, 1.003)	0.087		

Abbreviations: CHD, coronary heart disease; TC, total cholesterol; LDL, low-density lipoprotein; HDL, high-density lipoprotein; eGFR, estimated glomerular filtration rate; OR, odds ratio; CI, confidence interval. Notes: *p*-values < 0.05 are marked in bold.

## Data Availability

Data that support the findings of this study are available from the corresponding author on reasonable request.

## References

[1] Gutierrez J., Turan T.N., Hoh B.L., Chimowitz M.I. (2022). Intracranial atherosclerotic stenosis: Risk factors, diagnosis, and treatment. Lancet Neurol..

[2] Derdeyn C.P. (2018). Hemodynamics and oxygen extraction in chronic large artery steno-occlusive disease: Clinical applications for predicting stroke risk. J. Cereb. Blood Flow Metab..

[3] Wardlaw J.M., Smith C., Dichgans M. (2013). Mechanisms of sporadic cerebral small vessel disease: Insights from neuroimaging. Lancet Neurol..

[4] Wardlaw J.M., Smith E.E., Biessels G.J., Cordonnier C., Fazekas F., Frayne R., Lindley R.I., O’Brien J.T., Barkhof F., Benavente O.R. (2013). Neuroimaging standards for research into small vessel disease and its contribution to ageing and neurodegeneration. Lancet Neurol..

[5] Krasteva M.P., Lau K.K., Mordasini P., Tsang A.C.O., Heldner M.R. (2020). Intracranial Atherosclerotic Stenoses: Pathophysiology, Epidemiology, Risk Factors and Current Therapy Options. Adv. Ther..

[6] Li L., He S., Liu H., Pan M., Dai F. (2022). Potential risk factors of persistent postural-perceptual dizziness: A pilot study. J. Neurol..

[7] Du H., Chen C., Ye C., Lin F., Wei J., Xia P., Chen R., Wu S., Yuan Q., Chen H. (2020). Association between steno-occlusive middle cerebral artery and basal ganglia perivascular spaces. Front. Neurol..

[8] Park J.H., Kwon H.M., Lee J., Kim D.S., Ovbiagele B. (2015). Association of intracranial atherosclerotic stenosis with severity of white matter hyperintensities. Eur. J. Neurol..

[9] Zhai F.F., Yan S., Li M.L., Han F., Wang Q., Zhou L.X., Ni J., Yao M., Zhang S.-Y., Cui L.-Y. (2018). Intracranial arterial dolichoectasia and stenosis: Risk factors and relation to cerebral small vessel disease. Stroke.

[10] Levey A.S., Stevens L.A., Schmid C.H., Zhang Y.L., Castro A.F., Feldman H.I., Kusek J.W., Eggers P., Van Lente F., Greene T. (2009). A new equation to estimate glomerular filtration rate. Ann. Intern. Med..

[11] Wahlund L.O., Barkhof F., Fazekas F., Bronge L., Augustin M., Sjögren M., Wallin A., Ader H., Leys D., Pantoni L. (2001). A New Rating Scale for Age-Related White Matter Changes Applicable to MRI and CT. Stroke.

[12] van Swieten J.C., Hijdra A., Koudstaal P.J., van Gijn J. (1990). Grading white matter lesions on CT and MRI: A simple scale. J. Neurol. Neurosurg. Psychiatry.

[13] Doubal F.N., MacLullich A.M., Ferguson K.J., Dennis M.S., Wardlaw J.M. (2010). Enlarged Perivascular Spaces on MRI Are a Feature of Cerebral Small Vessel Disease. Stroke.

[14] Arba F., Leigh R., Inzitari D., Warach S.J., Luby M., Lees K.R. (2017). Blood-brain barrier leakage increases with small vessel disease in acute ischemic stroke. Neurology.

[15] Singer O.C., Berkefeld J., Nolte C.H., Bohner G., Reich A., Wiesmann M., Groeschel K., Boor S., Neumann-Haefelin T., Hofmann E. (2015). Collateral Vessels in Proximal Middle Cerebral Artery Occlusion: The endostroke Study. Radiology.

[16] Tan I., Demchuk A., Hopyan J., Zhang L., Gladstone D., Wong K., Martin M., Symons S., Fox A., Aviv R. (2009). CT Angiography Clot Burden Score and Collateral Score: Correlation with Clinical and Radiologic Outcomes in Acute Middle Cerebral Artery Infarct. Am. J. Neuroradiol..

[17] Sallustio F., Motta C., Pizzuto S., Diomedi M., Giordano A., D’Agostino V.C., Samà D., Mangiafico S., Saia V., Legramante J.M. (2017). CT angiography-based collateral flow and time to reperfusion are strong predictors of outcome in endovascular treatment of patients with stroke. J. NeuroInterventional Surg..

[18] Gupta A., Giambrone A., Gialdini G., Finn C.B., Delgado D., Gutierrez J., Wright C., Beiser A.B., Seshadri S., Pandya A. (2016). Abstract WP165: Silent Brain Infarction and Risk of Future Stroke: A Systematic Review and Meta-Analysis. Stroke.

[19] Fu J.H., Lu C.Z., Hong Z., Dong Q., Luo Y., Wong K.S. (2005). Extent of white matter lesions is related to acute subcortical infarcts and predicts further stroke risk in patients with first ever ischaemic stroke. J. Neurol. Neurosurg. Psychiatry.

[20] Vermeer S.E., Heijer T.D., Koudstaal P.J., Oudkerk M., Hofman A., Breteler M.M. (2003). Incidence and Risk Factors of Silent Brain Infarcts in the Population-Based Rotterdam Scan Study. Stroke.

[21] Baradaran H., Gialdini G., Mtui E., Askin G., Kamel H., Gupta A. (2016). Silent Brain Infarction in Patients with Asymptomatic Carotid Artery Atherosclerotic Disease. Stroke.

[22] Das A.S., Regenhardt R.W., Vernooij M.W., Blacker D., Charidimou A., Viswanathan A. (2019). Asymptomatic Cerebral Small Vessel Disease: Insights from Population-Based Studies. J. Stroke.

[23] Moroni F., Ammirati E., Magnoni M., D’Ascenzo F., Anselmino M., Anzalone N., Rocca M.A., Falini A., Filippi M., Camici P.G. (2016). Carotid atherosclerosis, silent ischemic brain damage and brain atrophy: A systematic review and meta-analysis. Int. J. Cardiol..

[24] Shi Y., Thrippleton M.J., Makin S.D., Marshall I., Geerlings M.I., de Craen A.J., van Buchem M.A., Wardlaw J.M. (2016). Cerebral blood flow in small vessel disease: A systematic review and meta-analysis. J. Cereb. Blood Flow Metab..

[25] Prabhakaran S., Liebeskind D.S., Cotsonis G., Nizam A., Feldmann E., Sangha R.S., Campo-Bustillo I., Romano J.G., on behalf of the MyRIAD Investigators (2021). Predictors of Early Infarct Recurrence in Patients with Symptomatic Intracranial Atherosclerotic Disease. Stroke.

[26] Baradaran H., Mtui E., Richardson J.E., Delgado D., Gupta A. (2017). Hemispheric Differences in Leukoaraiosis in Patients with Carotid Artery Stenosis: A Systematic Review. Clin. Neuroradiol..

[27] Ammirati E., Moroni F., Magnoni M., Rocca M.A., Messina R., Anzalone N., De Filippis C., Scotti I., Besana F., Spagnolo P. (2017). Relation between characteristics of carotid atherosclerotic plaques and brain white matter hyperintensities in asymptomatic patients. Sci. Rep..

[28] Caligiuri M.E., Perrotta P., Augimeri A., Rocca F., Quattrone A., Cherubini A. (2015). Automatic Detection of White Matter Hyperintensities in Healthy Aging and Pathology Using Magnetic Resonance Imaging: A Review. Neuroinformatics.

[29] Brown R., Benveniste H., Black S.E., Charpak S., Dichgans M., Joutel A., Nedergaard M., Smith K.J., Zlokovic B.V., Wardlaw J.M. (2018). Understanding the role of the perivascular space in cerebral small vessel disease. Cardiovasc. Res..

[30] Gouveia-Freitas K., Bastos-Leite A.J. (2021). Perivascular spaces and brain waste clearance systems: Relevance for neurodegenerative and cerebrovascular pathology. Neuroradiology.

[31] Troili F., Cipollini V., Moci M., Morena E., Palotai M., Rinaldi V., Romano C., Ristori G., Giubilei F., Salvetti M. (2020). Perivascular unit: This must be the place. The anatomical crossroad between the immune, vascular and nervous system. Front. Neuroanat..

[32] Shulyatnikova T., Hayden M.R. (2023). Why are perivascular spaces important?. Medicina.

[33] Shen M., Wei G., Cheng M., Jiang H. (2020). Association between enlarged perivascular spaces and internal carotid artery stenosis: A study in patients diagnosed by digital subtraction angiography. J. Stroke Cerebrovasc. Dis..

[34] Greenberg S.M., Vernooij M.W., Cordonnier C., Viswanathan A., Salman R.A.-S., Warach S., Launer L.J., Van Buchem M.A., Breteler M.M. (2009). Cerebral microbleeds: A guide to detection and interpretation. Lancet Neurol..

[35] Fisher M., French S., Ji P., Kim R.C. (2010). Cerebral microbleeds in the elderly: A pathological analysis. Stroke.

[36] Shoamanesh A., Kwok C., Benavente O. (2011). Cerebral Microbleeds: Histopathological Correlation of Neuroimaging. Cerebrovasc. Dis..

[37] Wang H.L., Zhang C.L., Qiu Y.M., Chen A.Q., Li Y.N., Hu B. (2021). Dysfunction of the blood-brain barrier in cerebral microbleeds: From bedside to bench. Aging Dis..

[38] Shu M.-J., Zhai F.-F., Zhang D.-D., Han F., Zhou L., Ni J., Yao M., Zhang S.-Y., Cui L.-Y., Jin Z.-Y. (2021). Metabolic syndrome, intracranial arterial stenosis and cerebral small vessel disease in community-dwelling populations. Stroke Vasc. Neurol..

[39] Liu J., Rutten-Jacobs L., Liu M., Markus H.S., Traylor M. (2018). Causal impact of type 2 diabetes mellitus on cerebral small vessel disease: A mendelian randomization analysis. Stroke.

[40] Evans L.E., Taylor J.L., Smith C.J., Pritchard H.A.T., Greenstein A.S., Allan S.M. (2021). Cardiovascular comorbidities, inflammation, and cerebral small vessel disease. Cardiovasc. Res..

[41] van Harten B., de Leeuw F.E., Weinstein H.C., Scheltens P. (2006). Brain imaging in patients with diabetes—A systematic review. Diabetes Care.

[42] Coll E., Botey A., Alvarez L., Poch E., Quintó L., Saurina A., Vera M., Piera C., Darnell A. (2000). Serum cystatin C as a new marker for noninvasive estimation of glomerular filtration rate and as a marker for early renal impairment. Am. J. Kidney Dis..

[43] Yao D., Li S., Jing J., Cai X., Jin A., Yang Y., Wang S., Meng X., Lin J., Mei L. (2022). Association of serum cystatin c with cerebral small vessel disease in community-based population. Stroke.

[44] Xu X., Ye X., Cai J., Gao T., Zhao G., Zhang W., Tong L., Gao F. (2018). Association of renal dysfunction with remote diffusion-weighted imaging lesions and total burden of cerebral small vessel disease in patients with primary intracerebral hemorrhage. Front. Aging Neurosci..

[45] Yang S., Cai J., Lu R., Wu J., Zhang M., Zhou X. (2017). Association between serum cystatin c level and total magnetic resonance imaging burden of cerebral small vessel disease in patients with acute lacunar stroke. J. Stroke Cerebrovasc. Dis..

[46] O’Rourke M.F., Safar M.E. (2005). Relationship between aortic stiffening and microvascular disease in brain and kidney: Cause and logic of therapy. Hypertension.

[47] Leng X., Leung T.W. (2023). Collateral flow in intracranial atherosclerotic disease. Transl. Stroke Res..

[48] Eker O.F., Rascle L., Cho T.-H., Mechtouff L., Derex L., Ong E., Berthezene Y., Nighoghossian N. (2019). Does small vessel disease burden impact collateral circulation in ischemic stroke treated by mechanical thrombectomy?. Stroke.

